# Escalated oxycodone self-administration is associated with expression of voltage gated and calcium activated potassium channels in the mesocorticolimbic system in rats

**DOI:** 10.3389/fphar.2025.1653356

**Published:** 2025-08-11

**Authors:** Ammanuel Y. Wabreha, Nasser Adjei, Bruce Ladenheim, Jean Lud Cadet, Atul P. Daiwile

**Affiliations:** Molecular Neuropsychiatry Research Branch, DHHS/NIH/NIDA Intramural Research Program, Baltimore, MD, United States

**Keywords:** oxycodone, potassium channels, mRNA, prefrontal cortex, nucleus accumbens, hippocampus, self-administration

## Abstract

**Background:**

The number of individuals diagnosed with opioid use disorder (OUD) has risen steeply because of increased prescribing of opioid drugs including oxycodone for chronic pain relief. When rats given extended access to oxycodone only a subset of animals self-administers more drug over time. Identifying the molecular mechanism associated with this behavior can introduce novel ways to combat OUD. Herein, we sought to identify the alteration in the expression of voltage gated and calcium activated potassium channels after extended access to oxycodone self-administration.

**Methods:**

We used male Sprague-Dawley rats that self-administered oxycodone for 20 days according to short-access (ShA, 3 h per day) and long-access (LgA, 9 h per day) paradigms.

**Results:**

LgA rats escalated their oxycodone intake and developed into two phenotypes, named long-access high (LgA-H, escalated intake) and long-access low (LgA-L, non-escalated intake) rats, based on the quantities of oxycodone intake during the self-administration experiment. ShA rats maintained similar oxycodone intake throughout 20 days of self-administration. Rats were euthanized 2 h after the last self-administration session and their prefrontal cortex (PFC), nucleus accumbens (NAc), and hippocampus (HIP) were dissected out for gene expression analysis. Given the relationship between potassium channels and substance use disorder we performed gene expression analysis for voltage and calcium activated potassium channels. The expression of potassium channels in oxycodone self-administered rats was found to be brain region dependent. Specifically, LgA-H rats displayed increased expression of *Kcnd2*, *Kcnd3*, *Kcng2* and *Kcnt1* in their NAc. In the PFC, LgA-L group showed higher mRNA levels for *Kcna3*, *Kcna4*, *Kcnd3*, *Kcnq4*, *Kcnq5*, *Kcnma1* and *Kcnn2*. Finally, *Kcna5*, *Kcna10*, *Kcng1*, *Kcnn1* and *Kcnn2* found to be upregulated in the HIP of ShA rats.

**Conclusion:**

Our observation is of significant translational importance providing further support that targeting potassium channel can lead to development of better therapeutic approaches against OUD in humans.

## 1 Introduction

The opioid epidemic remains a major public health crisis (Bergeria and Strain, 2022; Boscarino et al., 2010), despite efforts to reduce the overprescription of pain medications like oxycodone (King et al., 2011; Marie and Noble, 2023). Repeated oxycodone use often leads to tolerance and dependence among patients (Ellis et al., 2019; Kibaly et al., 2021), which can escalate to the misuse of more potent opioids, resulting in neuropsychiatric and neuropathological complications (Blackwood and Cadet, 2021; Cadet et al., 2014; Chamakalayil et al., 2024), and fatal overdoses (Sahebi-Fakhrabad et al., 2024; Tsang and Rodda, 2024). Oxycodone use disorder (OUD) is a biopsychosocial disorder in which someone loses control of drug taking even after the presence of adverse consequences (APA, 2024).

In 2024, the United States experienced a decline in overdose deaths according to data from the Centers for Disease Control and Prevention (CDC) which indicates a 14% decrease from the previous year (Ahmad FB et al., 2025). It is important to note that policy shifts that allowed over-the-counter naloxone sales and broadened Good Samaritan protections led to higher bystander intervention rates, played a measurable role in curbing opioid-related deaths. Meaning the public is likely to be abusing opioids at similar rates (Bohler et al., 2023). This decline represents the first substantial reduction in overdose fatalities in several years, validating the effectiveness of current research methods and offering hope for a potential reversal of the opioid epidemic’s trajectory. Ongoing efforts are essential to sustain and further this trend in reducing overdose fatalities.

Pharmacological treatments for OUD have traditionally focused on opioid receptor-related systems (Ali et al., 2024; Grande et al., 2023; Hayes et al., 2024; Olson et al., 2017). Advancing these treatments requires a deeper understanding of how repeated oxycodone use affects the human brain. Previously, we investigated this by utilizing an animal model that simulates key aspects of OUD and explore the molecular pathways impacted by oxycodone use (Blackwood and Cadet, 2021; Blackwood et al., 2019a; Salisbury et al., 2020). These studies from the past have confirmed a significant correlated relationship between potassium channels and substance use disorder (Cadet et al., 2017; Jayanthi et al., 2020). Potassium channels are important due to their roles in maintaining membrane potential, generating action potentials (Bean, 2007; Jan & January, 2012; Trimmer, 2015), facilitating neurotransmitter release (Klein et al., 1980), and supporting rhythmic neuronal firing (Noble, 1976; Solessio et al., 2000).

This current study focused on two specific subcategories of potassium channels, voltage gated, and calcium activated potassium channels. These potassium channels play crucial roles in regulating neuronal excitability and neurotransmitter release (Agarwal et al., 2025). Voltage-gated potassium channels (K_v_) are essential for action potential repolarization, thus influencing neuronal firing patterns (Shah and Aizenman, 2014). By facilitating the return of neurons to their resting state, K_v_ channels regulate synaptic activity, a critical role in the brains reward pathways (Ramirez-Navarro et al., 2024). There are eleven families in the K_v_ group, which are organized by their subunit composition, location in the cell and voltage threshold (Alfaro-Ruíz et al., 2019). Calcium-activated potassium channels (K_Ca_) are activated by intracellular calcium levels and contribute to modulating synaptic plasticity, a key process underlying learning, memory, and habit formation (Kuiper et al., 2012). There are three groups of K_Ca_: small conductance like *Kcnn1*, *Kcnn2* and *Kcnn3*, Large conductance like *Kcnma1*, and sodium activated like *Kcnt1* (Agarwal et al., 2025; McCoy et al., 2021). Small conductance K_Ca_ play an important role in synaptic plasticity and brain rhythmic activity (Sun et al., 2020).

Irregularities in either type of potassium channel can disrupt normal neuronal signaling, enhancing reward-related behaviors and increasing vulnerability to substance abuse (Alam et al., 2023). Previous studies suggest that impaired potassium channel function may alter dopamine release, which in turn alters excitability within the brain’s reward circuitry, contributing to the development and persistence of addictive behaviors (Blackwood et al., 2019b; Daiwile et al., 2022; Pignatelli and Bonci, 2015; Shah and Aizenman, 2014). To further understand the relationship between potassium channels and OUD, we examined transcriptional changes in the prefrontal cortex (PFC), nucleus accumbens (NAc) and the hippocampus (Hip) in rats that self-administered small or large amounts of oxycodone over a 20-day period.

## 2 Materials and methods

### 2.1 Subjects

Male Sprague Dawley rats, weighing between 350 and 400 g were procured from Charles River, Kingston, NY, United States. The rats were housed in a controlled setting with a reversed 12-h light/dark cycle with free access to food and water. All self-administration sessions began (∼9:00 a.m. everyday) at the start of the dark phase of the light/dark cycle. All experimental procedures adhered to the guidelines outlined in the National Institutes of Health (NIH) Guide for the Care and Use of Laboratory Animals and were approved by the NIDA (National Institute of Drug Abuse) Animal Care and Use Committee at the Intramural Research Program (IRP).

### 2.2 Intravenous surgery

Rats were first anesthetized using a combination of ketamine (50 mg/kg) and xylazine (5 mg/kg). A polyurethane catheter was surgically inserted into the right jugular vein while the external end was mounted to the back of the rat (Blackwood et al., 2019a). The rats were then given a recovery period of 7 days before beginning self-administration training.

### 2.3 Oxycodone self-administration

Following an established protocol, drug-naive rats were trained to self-administer oxycodone (0.1 mg/kg/infusion) or saline within a sound-attenuated chamber using a FR1 schedule (Blackwood et al., 2019b). A total of 36 rats were divided into three groups: Saline (Sal) (n = 8), Short-access (ShA) (n = 10), or Long-access (LgA) (n = 18). Short-access rats were allowed to self-administer oxycodone for only one 3-h session for the entirety of the study (days 1–20). Long-access and saline rats were scheduled to self-administer for three sessions using, one 3-h session during days 1–5, followed by two 3-h sessions during days 6–10 and then three 3-h sessions for the rest of the study (days 11–20). The 20-day timeframe was selected based on previous paradigms in the literature that reliably produce escalation and allow for the emergence of compulsive-like drug intake behaviors (Blackwood and Cadet, 2021; Blackwood et al., 2019a; Blackwood et al., 2019b).

We gradually increased access to oxycodone over 4 weeks to prevent any adverse effects of oxycodone intake. There was a 20 s timeout between each infusion. Each 3-h session for LgA and Sal from day 6 to day 20 was separated by a 30-min timeout. This 30-min break was implemented to prevent overdoses as there was no limit to the number of infusions a rat could take during a session. We also included a 48h weekend abstinence period between every 5 days of SA to prevent significant weight loss that might have led to the elimination of some rats from the study. Catheter patency was tested thought the experiment. Rats were euthanized 2 hours after the first session of the last day. Saline animals underwent similar surgical procedures as oxycodone rats, were placed in the identical operant chambers, and experienced similar cue presentations during SA sessions.

### 2.4 RNA extraction and cDNA conversion

Two hours after the final self-administration session, rats were euthanized via rapid decapitation using a guillotine. PFC, NAc and HIP tissues were dissected using precise neuroanatomical coordinates using the Atlas (Paxinos and Watson, 2006) and then immediately snap-frozen on dry ice before being stored at −80 °C (Blackwood et al., 2019b). Total RNA was extracted from tissue using Qiagen RNeasy Mini kit (Qiagen, Valencia, CA, United States). A half microgram (0.5 μg) of total RNA was reverse-transcribed to cDNA with oligo dT primers using Advantage RT-for-PCR kit (Clontech, Mountain View, CA, United States).

### 2.5 Quantitative RT-PCR

qRT-PCR was carried out using a Roche LightCycler 480 II (Roche Diagnostics, Indianapolis, IN) with Luna Universal qPCR SYBR GREEN (NEB Inc, Ipswich, MA) following the manufacturer’s protocol. We purchased gene-specific primers from Integrated DNA Technologies (IDT) (Coralville, IA, United States). These primers were designed using Thermo Fisher Scientific (OligoPrefect Primer Designer software). We normalized mRNA using beta-2 microglobulin (B2M), Clathrin, and ornithine decarboxylase antizyme (OAZ1) as reference genes and the mRNA expression of target genes were reported as fold changes. The primer sequences used for PCR are listed in Supplementary Table S1.

### 2.6 Statistical analyses

Behavioral data were analyzed with the statistical program GraphPad Prism 10 using factorial ANOVA with repeated measures. Independent variables were the rat reward types (Sal, ShA, LgA-L, LgA-H), within-subject factor SA day (training days 1–20). Oxycodone intake served as the dependent variable. A second-degree polynomial regression model was used to identify potential non-linear patterns in oxycodone intake over 20 days of SA for individual animals, to segregate the rats into LgA-H and LgA-L subgroups. The rats which escalated their intake were termed as LgA-H where those who did not were termed as LgA-L. Biochemical data were analyzed using one-way ANOVA followed by the Tukey’s multiple comparisons test if the main effect was significant. The slopes of all the regression lines were calculated using one-way ANOVA. Statistical significance for all hypothesis tests was set at p < 0.05.

## 3 Results

### 3.1 Some rats exposed to LgA oxycodone self-administration escalated their drug intake

**FIGURE 1:**
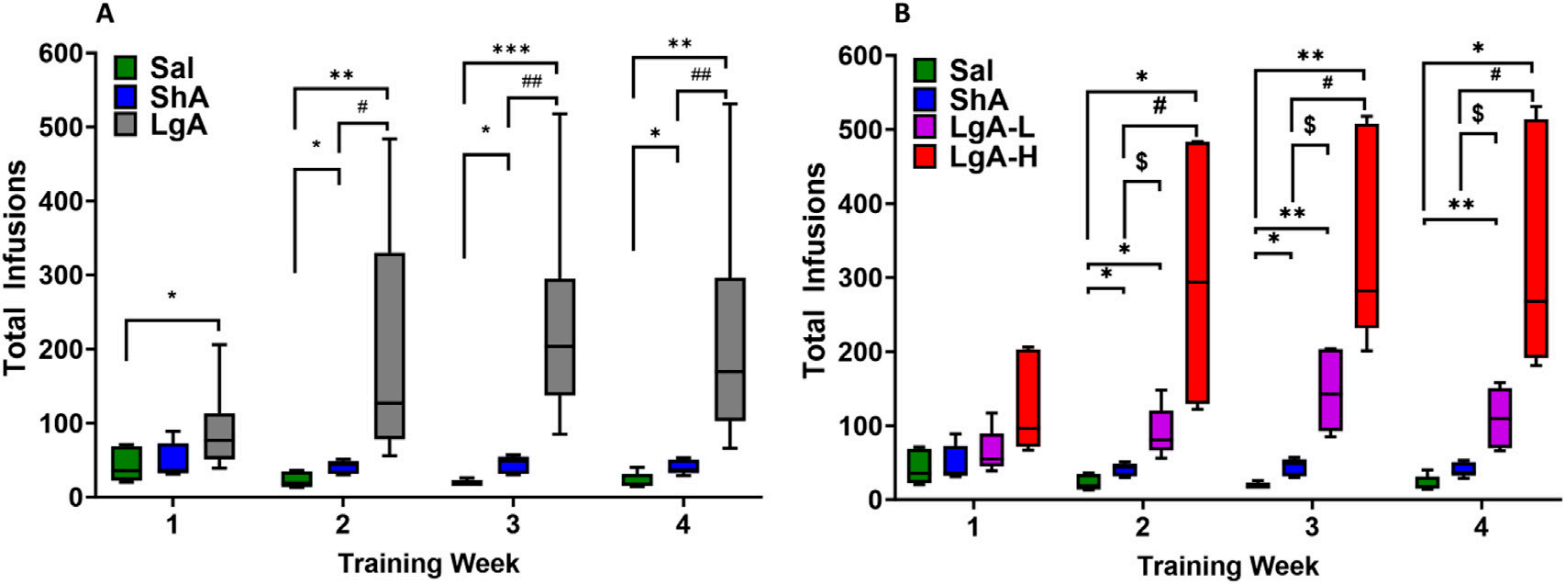
Insights into Oxycodone Self-Administration: Behavioral Data. **(A)** Total infusions by Sal (8), LgA (18) and ShA (10) groups. **(B)** LgA rats show two distinct intake phenotypes, high (LgA-H) (n = 11) and low (LgA-L) (n = 7) based on their drug intake. Key to statistics: *, **, *** = p < 0.05, 0.01, 0.001, LgA-H, LgA-L, or ShA in comparison to saline rats; #, ##, = p < 0.05, 0.01 when comparing LgA-H/LgA rats to ShA rats; $, = p < 0.05, when comparing LgA-L rats to ShA rats.

As shown previously (Blackwood et al., 2019a) we analyzed the behavioral data using repeated measures two-way ANOVA with groups (ShA vs. LgA) and training weeks as factors. We observed significant effects for group [F_(1, 49)_ = 37.6, p < 0.0001], training week [F_(1.994, 97.71)_ = 26.56, p < 0.0001], and group × training week [F_(3, 147)_ = 23.51, p < 0.0001]. Post-hoc test showed LgA rats had greater total oxycodone intake than ShA and Sal rats (Figure 1A).

Interestingly, not all LgA rats self-administered oxycodone to the same degree. We performed regression analyses to compare oxycodone acquisition and rate of change of oxycodone intake over time (Blackwood et al., 2019b). We found that some LgA rats significantly escalated their oxycodone intake over the period of 20 days, whereas others did not escalate their intake. Animals that escalated their oxycodone intake over 20 days of SA were called Long-access High (LgA-H), whereas those that did not escalate were named Long-access Low (LgA-L) (Figure 1B). We reanalyzed the behavioral data with four phenotypes LgA-H, LgA-L, ShA, and Sal. Two-way ANOVA showed significant effects for groups [F_(2, 48)_ = 86.30, p < 0.0001], oxycodone intake [F_(2.594, 124.5)_ = 82.67, p < 0.0001], and group × oxycodone intake interaction [F_(6, 144)_ = 34.17, p < 0.0001] (Figure 1B).

Previous studies reasoned that the differences at the behavioral level may be due to different drug-induced molecular neuroadaptations in potassium channel expression between the different phenotypes (McCoy et al., 2021). Building on these findings, we sought to investigate the molecular mechanisms underlying potassium channel differences to better understand their role in OUD vulnerability.

### 3.2 Prefrontal cortex (PFC)

**FIGURE 2:**
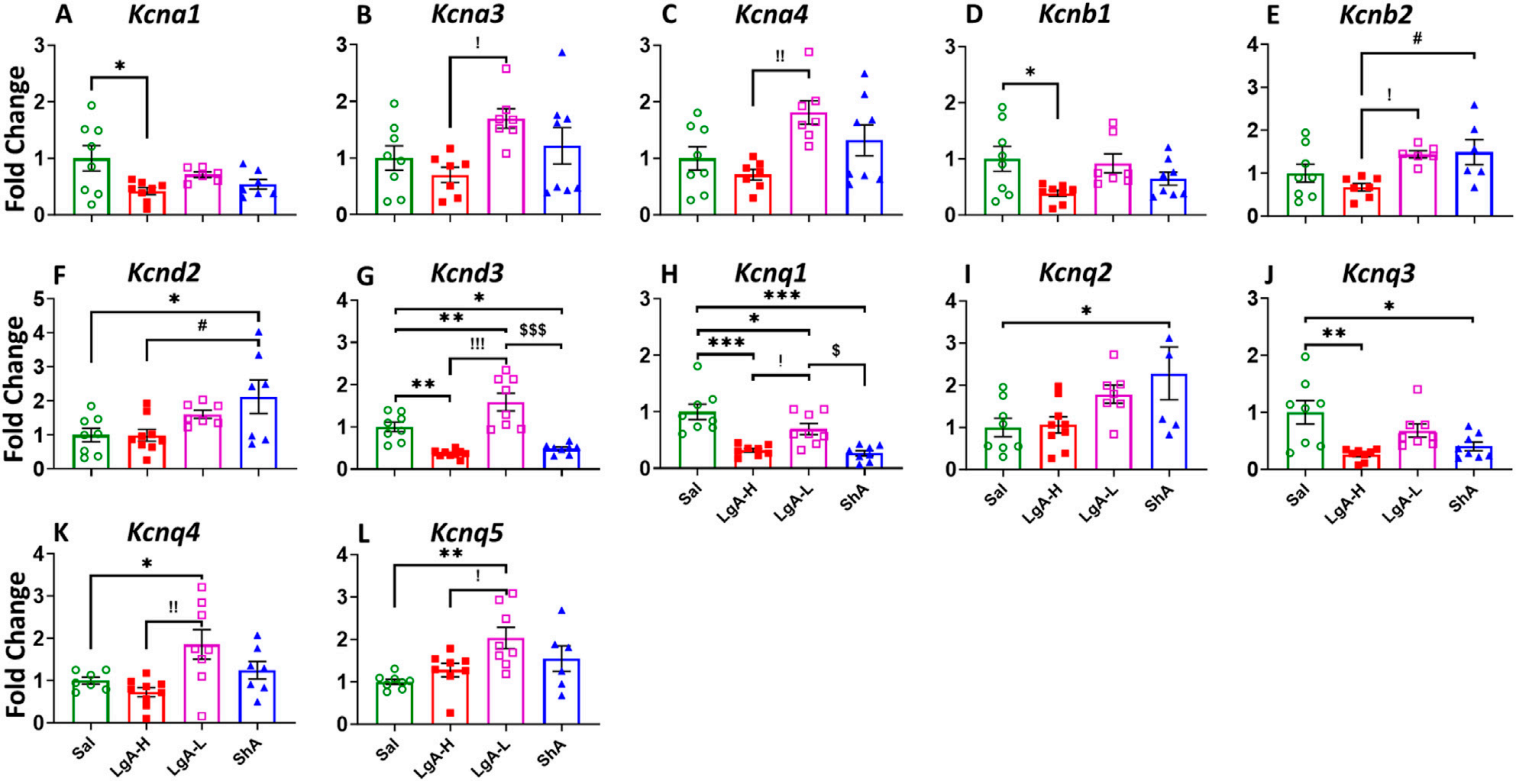
Voltage gated potassium channels showed increased expression in Prefrontal Cortex of LgA-L rats. **(A)** *Kcna1*, **(B)** *Kcna3,* **(C)** *Kcna4,* **(D)** *Kcnb1,* **(E)** *Kcnb2,* **(F)** *Kcnd2,* **(G)** *Kcnd3,* **(H)** *Kcnq1,* **(I)** *Kcnq2,* **(J)** *Kcnq3,* **(K)** *Kcnq4,* and **(L)** *Kcnq5.* Key to statistics: *, *** = p < 0.05, 0.001, LgA-H, LgA-L, or ShA in comparison to saline rats; #, ##, ### = p < 0.05, 0.01, 0.001, when comparing LgA-H rats to ShA rats; $, $$ = p < 0.05, 0.01, when comparing LgA-L rats to ShA rats. !, !!, !!! = p < 0.05, 0.01, 0.001, when comparing LgA-H rats to LgA-L rats.

The ANOVA for voltage gated potassium channels (K_v)_ in the PFC showed significant effects of treatment group on *Kcna1* [F_(3, 25)_ = 3.612, p = 0.0271], *Kcna3* [F_(3, 26)_ = 3.097, p = 0.0442], *Kcna4* [F_(3, 26)_ = 4.735, p = 0.0091], *Kcnb1* [F_(3, 27)_ = 3.344, p = 0.0338], *Kcnb2* [F_(3, 23)_ = 4.037, p = 0.0192], *Kcnd2* [F_(3, 27)_ = 4.028, p = 0.0172], *Kcnd3* [F_(3, 29)_ = 22.43, p < 0.0001], *Kcnq1* [F_(3, 28)_ = 14.87, p < 0.0001], *Kcnq2* [F_(3, 26)_ = 3.615, p = 0.0264], *Kcnq3* [F_(3, 28)_ = 6.843, p = 0.0013], *Kcnq4* [F_(3, 27)_ = 5.265, p = 0.0054], and *Kcnq5* [F_(3, 26)_ = 5.099, p = 0.0066] (Figures 2A–L). For *Kcna1* and *Kcnb1,* LgA-H rats revealed decreased mRNA expression compared to Sal (Figures 2A,D). LgA-L rats showed elevated mRNA levels for *Kcna3* and *Kcna4* when compared to LgA-H (Figures 2B,C). Likewise LgA-L rats displayed increased expression of *Kcnq4*, and *Kcnq5* when compared to Sal and LgA-H (Figures 2K,L). LgA-L rats and ShA rats exhibited higher expression of *Kcnb2* compared to the LgA-H phenotype (Figure 2E). Only ShA animals unveiled increased *Kcnd2* expression when compared to Sal and LgA-H rats (Figure 2F), while for *Kcnq3* this increase was only if compared to the Sal phenotype (Figure 2J). *Kcnd3*’s mRNA level was found to be decreased among LgA-H and ShA rats when compared to Sal, whereas LgA-L rats showed increased expression compared to Sal, LgA-H and ShA (Figure 2G). The mRNA level of *Kcnq1* was found to be decreased among LgA-H, LgA-L and ShA, in addition there was a significant difference between the magnitude of LgA-L’s downregulation and that of LgA-H and ShA (Figure 2H). We also observed increased expression of *Kcnq2* in the PFC of ShA rats than Sal (Figure 2I). No significance was found for *Kcna2, Kcna5, Kcna6, Kcnd1, Kcng1, Kcng2, Kcng3,* and *Kcng4* (Supplementary Figure S1A–H).

**FIGURE 3:**
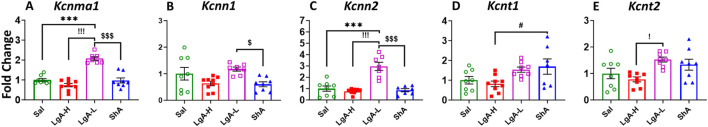
Calcium activated potassium channels showed increased expression in Prefrontal Cortex of LgA-L. **(A)** *Kcnma1*, **(B)** *Kcnn1,* **(C)** *Kcnn2,* **(D)** *Kcnt1,* and **(E)** *Kcnt2.* Key to statistics: *, *** = p < 0.05, 0.001, LgA-H, LgA-L, or ShA in comparison to saline rats; #, ##, ### = p < 0.05, 0.01, 0.001, when comparing LgA-H rats to ShA rats; $, $$ = p < 0.05, 0.01, when comparing LgA-L rats to ShA rats. !, !!, !!! = p < 0.05, 0.01, 0.001, when comparing LgA-H rats to LgA-L rats.

We also analyzed the results for calcium-activated potassium channels (K_Ca_), ANOVA revealed significant effects for *Kcnma1* [F_(3, 28)_ = 34.98, p < 0.0001], *Kcnn1* [F_(3, 28)_ = 3.923, p = 0.0186], *Kcnn2* [F_(3, 26)_ = 20.96, p < 0.0001], *Kcnt1* [F_(3, 28)_ = 3.65, p = 0.0244], and *Kcnt2* [F_(3, 28)_ = 4.493, p = 0.0107] (Figures 3A–E). LgA-L revealed increased mRNA expression for *Kcnma1* and *Kcnn2* in their PFC when compared to Sal, LgA-H and ShA rats (Figures 3A,C). LgA-L also displayed higher expression then LgA-H for *Kcnt2* (Figure 3E). Interestingly ShA showed decreased mRNA level for *Kcnn1* when compared with LgA-L (Figure 3B) and increased expression for *Kcnt1* when compared to LgA-H rats (Figure 3D).

### 3.3 Nucleus accumbens (NAc)

**FIGURE 4:**
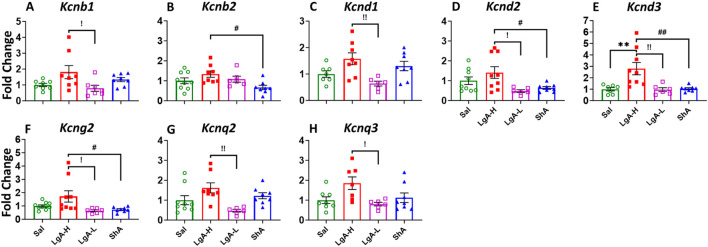
Voltage gated potassium channels showed increased expression in Nucleus Accumbens of LgA-H rats. **(A)** *Kcnb1*, **(B)** *Kcnb2,* **(C)** *Kcnd1,* **(D)** *Kcnd2,* **(E)** *Kcnd3,* **(F)** *Kcng2,* **(G)** *Kcnq2,* and **(H)** *Kcnq3.* Key to statistics: *, *** = p < 0.05, 0.001, LgA-H, LgA-L, or ShA in comparison to saline rats; #, ##, ### = p < 0.05, 0.01, 0.001, when comparing LgA-H rats to ShA rats; $, $$ = p < 0.05, 0.01, when comparing LgA-L rats to ShA rats. !, !!, !!! = p < 0.05, 0.01, 0.001, when comparing LgA-H rats to LgA-L rats.

We observed a significant one-way ANOVA results in the rats NAc for *Kcnb1* [F_(3, 27)_ = 3.268, p = 0.0365], *Kcnb2* [F_(3, 27)_ = 3.785, p = 0.0218], *Kcnd1* [F_(3, 25)_ = 5.122, p = 0.0067], *Kcnd2* [F_(3, 28)_ = 4.043, p = 0.0166], *Kcnd3* [F_(3, 26)_ = 7.580, p = 0.0008], *Kcng2* [F_(3, 29)_ = 4.037, p = 0.0163], *Kcnq2* [F_(3, 27)_ = 4.868, p = 0.0078], and *Kcnq3* [F_(3, 25)_ = 3.841, p = 0.0217] (Figures 4A–H). Moreover, LgA-H rats revealed a significant increase for *Kcnb1*, *Kcnd1*, *Kcnq2*, and *Kcnq3* when compared to LgA-L (Figures 4A,C,G,H) and exhibited higher *Kcnb2* expression when compared with ShA (Figure 4B). Higher mRNA level for *Kcnd2* and *Kcng2* was also seen in LgA-H rats than LgA-L and ShA rats (Figures 4D,F). Likewise, only LgA-H rats had higher expression of *Kcnd3* than Sal, LgA-L and ShA rats (Figure 4E). No significance changes were found for *Kcna1, Kcna2, Kcna3, Kcna4, Kcna5, Kcna6, Kcna10, Kcng1, Kcng3, Kcng4, Kcnq1, Kcnq4,* and *Kcnq5* (Supplementary Figure S2A–M).

**FIGURE 5:**
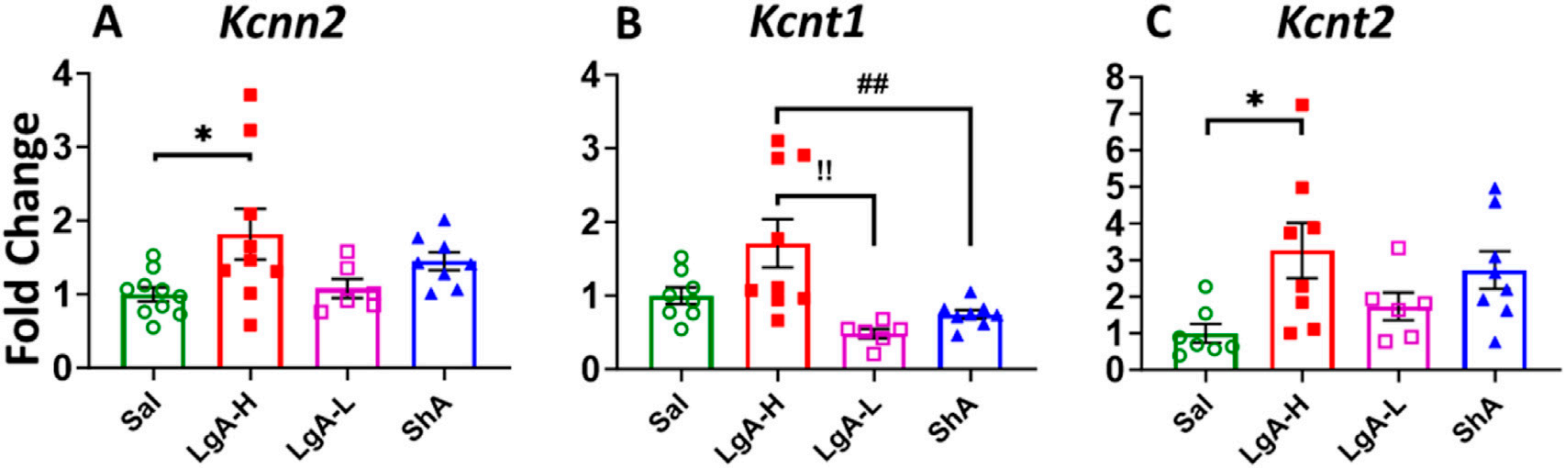
Calcium activated potassium channels showed increased expression in Nucleus Accumbens of LgA-H rats. **(A)** *Kcnn2*, **(B)** *Kcnt1,* and **(C)** *Kcnt2.* Key to statistics: *, *** = p < 0.05, 0.001, LgA-H, LgA-L, or ShA in comparison to saline rats; #, ##, ### = p < 0.05, 0.01, 0.001, when comparing LgA-H rats to ShA rats; $, $$ = p < 0.05, 0.01, when comparing LgA-L rats to ShA rats. !, !!, !!! = p < 0.05, 0.01, 0.001, when comparing LgA-H rats to LgA-L rats.

Like that of K_v_, K_Ca_ revealed significant effects of treatment group on *Kcnn2* [F_(3, 29)_ = 3.398, p = 0.0309], *Kcnt1* [F_(3, 27)_ = 6.787, p = 0.0015] and *Kcnt2* [F_(2, 25)_ = 3.499, p = 0.0302] (Figures 5A–C). mRNA expression levels of *Kcnn2* and *Kcnt2* was higher in LgA-H animals when compared to Sal (Figures 5A,C). LgA-H rats also revealed a higher level for *Kcnt1* when compared with LgA-L and ShA rats (Figure 5C). No changes were seen for *Kcnn1* (Supplementary Figure S3A).

### 3.4 Hippocampus (HIP)

**FIGURE 6:**
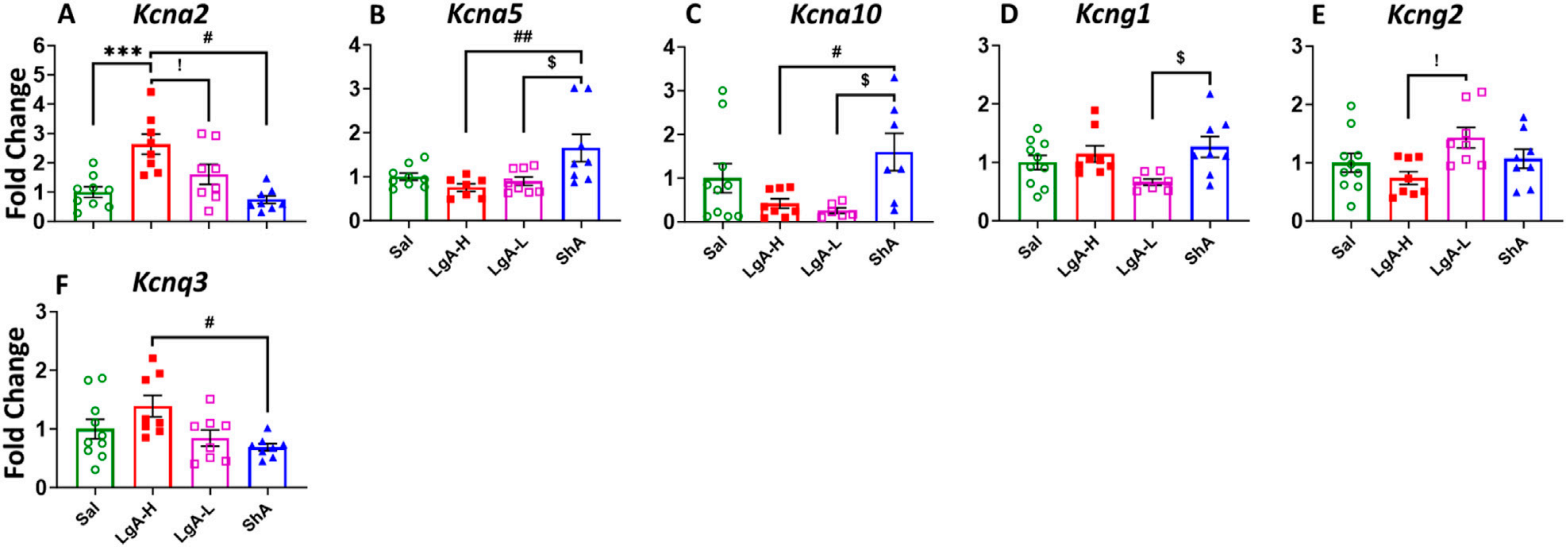
Voltage gated potassium channels showed increased expression in Hippocampus of ShA rats. **(A)** *Kcna2*, **(B)** *Kcna5,* **(C)** *Kcna10,* **(D)** *Kcng1,* **(E)** *Kcng2,* and **(F)** *Kcnq3.* Key to statistics: *, *** = p < 0.05, 0.001, LgA-H, LgA-L, or ShA in comparison to saline rats; #, ##, ### = p < 0.05, 0.01, 0.001, when comparing LgA-H rats to ShA rats; $, $$ = p < 0.05, 0.01, when comparing LgA-L rats to ShA rats. !, !!, !!! = p < 0.05, 0.01, 0.001, when comparing LgA-H rats to LgA-L rats.

When looking at K_v_ in the HIP we found a significant ANOVA effect of treatment group on *Kcna2* [F_(3, 29)_ = 10.08, p = 0.0001], *Kcna5* [F_(3, 28)_ = 5.066, p = 0.0063], *Kcna10* [F_(3, 27)_ = 3.778, p = 0.0220], *Kcng1* [F_(3, 29)_ = 3.342, p = 0.0328], *Kcng2* [F_(3, 30)_ = 3.132, p = 0.0401], and *Kcnq3* [F_(3, 30)_ = 3.834, p = 0.0195] (Figures 6A–F). Further LgA-H rats displayed significant increase in the expression of *Kcna2* compared to Sal, LgA-L and ShA (Figure 6A) and for Kcnq3 then ShA (Figure 5F). Whereas ShA animals revealed higher mRNA level for *Kcna5* and *Kcna10* than LgA-H and LgA-L (Figures 5B,C) and for *Kcng1* only when compared to LgA-L (Figure 5D). While LgA-L phenotype revealed elevated mRNA level for *Kcng1* when compared with LgA-H rats (Figure 5E). No significance difference was found for *Kcna1, Kcna3, Kcna6, Kcnb1, Kcnb2, Kcnd1, Kcngd2, Kcnd3, Kcng3, Kcng4, Kcnq1, Kcnq2, Kcnq4,* and *Kcnq5* (Supplementary Figure S4A–N).

**FIGURE 7:**
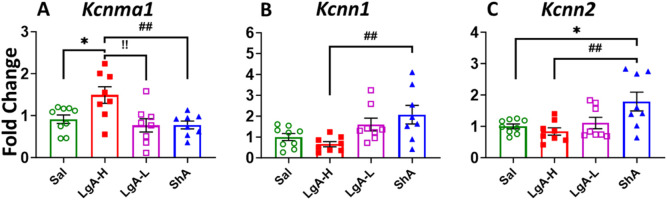
Calcium activated potassium channels showed increased expression in Hippocampus of ShA rats. **(A)** *Kcna1*, **(B)** *Kcnn1,* and **(C)** *Kcnn2.* Key to statistics: *, *** = p < 0.05, 0.001, LgA-H, LgA-L, or ShA in comparison to saline rats; #, ##, ### = p < 0.05, 0.01, 0.001, when comparing LgA-H rats to ShA rats; $, $$ = p < 0.05, 0.01, when comparing LgA-L rats to ShA rats. !, !!, !!! = p < 0.05, 0.01, 0.001, when comparing LgA-H rats to LgA-L rats.

Moreover, we also saw a significant effect for *Kcnma1* [F_(3, 30)_ = 5.024, p = 0.0061], *Kcnn1* [F_(3, 29)_ = 4.851, p = 0.0074], and *Kcnn2* [F_(3, 29)_ = 5.134, p = 0.0057] (Figures 7A–C). ShA rats displayed significant higher mRNA levels for *Kcnn1* and *Kcnn2* than LgA-H rats (Figures 7B,C). Interestingly, expression of *Kcnma1* mRNA found to be significantly increased in the LgA-H group compared to Sal, LgA-L and ShA (Figure 7A). No significance was found for *Kcnt1, Kcnt2* and *Kcnn3* (Supplementary Figure S5A–C).

In the present study, we observed significant differences among the experimental groups, including: (1) LgA-H compared to LgA-L and/or ShA; (2) LgA-L compared to LgA-H and/or ShA; and (3) ShA compared to LgA-H and/or LgA-L. These differences may reflect inherent phenotypic variability or adaptive processes not directly attributable to the self-administration (SA) procedure. Rather than representing direct drug-induced molecular changes, such variability could result from pre-existing individual differences or secondary effects of prolonged drug exposure and behavioral stratification. Therefore, we propose that future studies should not limit comparisons to control groups alone but also include contrasts among drug-exposed groups to better capture the contribution of intrinsic variability and adaptive mechanisms to substance use disorder.

## 4 Discussion

The present study assessed potential changes in the expression of voltage-gated and calcium-activated potassium channels in the mesocorticolimbic projection areas of rats who were allowed to self-administer oxycodone for 20 days. We observed a difference in drug intake behavior among rats with long access (LgA) to oxycodone. LgA rats took more infusions than ShA rats and post facto divided into two oxycodone self-administering phenotypes: LgA-H and LgA-L. Our current behavior observations of high and low self-administration by LgA rats align with previously published studies for methamphetamine (METH) (Daiwile et al., 2021; Daiwile et al., 2019; Daiwile et al., 2022), cocaine (de Guglielmo et al., 2024) and oxycodone (Blackwood and Cadet, 2021; Blackwood et al., 2019a; Blackwood et al., 2019b). Moreover, repeated oxycodone use has been reported in tolerance development among patients (Ellis et al., 2019; Kibaly et al., 2021) resulting in higher drug use to achieve comparable effects. Likewise in the present study, LgA-H rats may have developed tolerance earlier than LgA-L rats, potentially contributing to their higher oxycodone intake. Evidence reviewed by McCoy et al. (2021) implicates the involvement of potassium channels in substance use disorders including METH (Cadet et al., 2017), alcohol (Rinker et al., 2017) and cocaine (McCall et al., 2017). Prior studies, using RNA sequencing also identified that oxycodone exposure can alter potassium channel expression and, be associated with different behavioral patterns (McCall et al., 2017). We believed that the observed behavior is due to alterations in the expression of potassium channels in the PFC, NAc and Hip. Moreover, these brain structures receive projections from the VTA which plays an important role in reward processing (Khayat and Yaka, 2024). Our study identified a brain region dependent difference in the expression of potassium channels in oxycodone self-administered rats. Rats that self-administered the most oxycodone (LgA-H) revealed a significant increase in the expression of both voltage gated and calcium activated potassium channels in their NAc. In contrast, LgA-L phenotypes increased in both voltage gated and calcium activated potassium channels in their PFC. We also identified a significant increase of potassium channels in the hippocampus of ShA rats.

### 4.1 Activation of potassium channels in the NAc of LgA-H rats

Rats that took the highest levels of oxycodone, LgA-H, were suspected to be the most vulnerable to OUD. They showed a very interesting expression pattern for potassium channels in their NAc. The Nucleus Accumbens is a key hub for reward circuitry involved in drug-taking behaviors (Kuhn et al., 2014), and can enhance the reinforcing effects of opioids (Cornish et al., 1999). It also plays a significant role in processing pleasurable experiences like eating, drug use, and social interactions (Bassareo and Di Chiara, 1999; Carelli, 2002; Olsen, 2011). Potassium channels in the NAc regulate reward behavior by influencing synaptic plasticity (Fernández-Fernández and Lamas, 2021; Kim and Hoffman, 2008). Potassium channels help control inhibitory signaling within the brain (Qiu et al., 2025), which is crucial for suppressing impulsive behaviors. Dysfunction in potassium channel activity can lead to hyperexcitability of neurons, heightening drug-cue reactivity and impairing self-regulation (Humphries and Dart, 2015; Wu et al., 2024). The symptoms you can expect to see because of this dysregulation are irregular heartbeats (Fanoe et al., 2009; Meents et al., 2018) respiratory depression (Montandon et al., 2016; Wei and Ramirez, 2019) and antinociceptive affects (Nakamura et al., 2014). Dysregulation of potassium channels can also lead to seizures (Gross et al., 2016; Zhang et al., 2021), autism (Liu et al., 2022), and ataxia (Pollini et al., 2020).

In the NAc, LgA-H rats revealed consistent upregulation of potassium channels, when compared to LgA-L rats, supporting the link between potassium channel expression and increased vulnerability to OUD. There was higher level of *Kcnd2*, *Kcnd3*, *Kcng2* and *Kcnt1* in NAc of LgA-H rats compared to ShA and LgA-L and expression of *Kcnn2* and *Kcnt2* was different then Sal. Moreover, administration of a potassium channel inhibitor in the NAc of rats was found to attenuate cocaine seeking behaviors in them (Xia et al., 2024), further supporting their role in addiction-related neuroadaptations. Additionally, increased potassium channels in the NAc would cause a hyperexcitable reward circuit making it easier to succumb to oxycodone abuse (Yuferov et al., 2018). These findings suggest that reducing the frequency of potassium channels in the NAc, could serve as a treatment for OUD.

### 4.2 Activation of potassium channels in the PFC of LgA-L rats

We thought studying molecular neuroadaptations in LgA-L rats might be interesting because they failed to increase their oxycodone intake despite having the same extended access as the LgA-H group. LgA-L phenotypes displayed higher mRNA levels for *Kcna3*, *Kcna4*, *Kcnd3*, *Kcnq4*, *Kcnq5*, *Kcnma1* and *Kcnn2* in their PFC. The PFC is a brain region of significant interest for its role in decision making, cognitive function (Bausch et al., 2015; Yu et al., 2016; Yu et al., 2019), and impulse control (Arnsten, 2009; Ramnani and Owen, 2004). The PFC, along with subcortical circuits, also plays a crucial role in self-control, and social behavior, influencing both drug-cue reactivity and the regulation of craving and drug-seeking in substance use disorders (Daiwile et al., 2022; Jasinska et al., 2015). Potassium channels control the flow of potassium ions across cell membranes, which is essential for maintaining the resting membrane potential. This means they can play a significant role in regulating self-control and decision-making, by influencing the excitability of neurons (Greene and Hoshi, 2017; Humphries and Dart, 2015). By stabilizing neuronal excitability, potassium channels play a vital role in maintaining balanced neural activity, necessary for recovery in individuals with substance use disorders.

The results we found in the PFC contrasts the pattern we saw in the NAc, where upregulated potassium channel expression was associated with LgA-H rats. The connection between high potassium channel expression in the PFC and low intake behavior suggest the presence of a compensatory mechanism that can reduce a subject’s susceptibility to OUD. Previous studies have shown that enhanced potassium channel activity in the PFC is linked to reduced compulsive drug use (Buchta and Riegel, 2015). Studies on the alcohol consumption in *drosophila* (Cavaliere et al., 2012), rodents (Padula et al., 2015), and humans (Clarke et al., 2011) have found K_Ca_ and K_v_ levels to be decreased in compulsive animals (Cannady et al., 2018), meaning increasing their levels may decrease OUD susceptibility. Other studies show that acute or chronic drug exposure can decrease potassium channel expression in the PFC (Dong et al., 2005). Taken together, these results show that increased expression of potassium channels in the PFC might lead to a decreased oxycodone intake in LgA-L rats. More importantly, these discoveries highlight the PFC as another fundamental brain region where potassium channel modulation plays a role in the neurobiology of OUD (Padula et al., 2015) and suggest that targeting potassium channels may be an advantageous therapeutic approach.

### 4.3 Activation of potassium channels in the hip of ShA rats

Lastly, we investigated neuro-molecular changes in the brains of ShA rats, as this group can give us insights into molecular adaptations that are due to the mere exposure of oxycodone. The results show multiple K_v_ (*Kcna5*, *Kcna10*, *Kcng1*) and K_Ca_ (*Kcnn1*, *Kcnn2*) to be upregulated in the Hip of ShA rats. The hippocampus is responsible for the rats’ abilities in learning and memory (O'Dell et al., 2015), functions that we know are impacted by oxycodone intake (Cherrier et al., 2009). Specifically, the VTA to hippocampus neuronal projection plays a vital role in the primary reward circuit and is even more essential for memory formation (Atweh and Kuhar, 1977; Bird and Burgess, 2008). Potassium channels contribute to these functions by maintaining the balance between excitation and inhibition in this circuit (Tsuboi et al., 2024). By maintaining proper neuronal excitability, they ensure that signals associated with reward experiences are accurately processed. Dysfunction in this system will contribute to impaired reward learning (Faulkner et al., 2024), potentially influencing behaviors seen in substance use disorder. This result is important to note as these same genes were not seen to be impacted in other brain regions by any behavioral group, indicating that their activation is linked to a protective neural state. This implies a connection between these genes and an early neurobiological response that reduces vulnerability to compulsive drug-seeking behavior. Moreover, the absence of these changes in LgA-H rats implies that the loss of this protective mechanism contributes to addiction progression.

## 5 Conclusion

In conclusion, we observed two phenotypes among LgA rats which are LgA-H and LgA-L, based on their oxycodone intake. We also found brain region-specific changes in the mRNA expression of voltage-gated and calcium-activated potassium channels in the PFC, NAc, and HIP. We suggest that the activation of Kv and KCa channel in the NAc of LgA-H rats might result in reduced excitability of neurons involved in reward circuit, thus influencing oxycodone taking behaviors. Alternatively, these changes might serve compensatory functions in that circuit. Unexpectedly, whereas LgA-L rats showed increased expression of potassium channel in their PFC, changes in the HIP were found in the ShA phenotypes. Together, these observations suggest potential important relationships between potassium channels in mesocorticolimbic systems and behavioral responses associated with oxycodone intake. Our results further support the notion that more efforts need to be spent to identify potential roles that brain regional differences might play in the clinical manifestations of oxycodone use disorder. Our findings also have important implications for potential treatment strategies, as they further support the therapeutic potential of potassium channel inhibitors or agonists. Although potassium channel represents a promising target, brain region-specific modulation within functionally diverse circuits poses significant challenges and need to take in account when developing therapeutic application. Future studies should focus on subregional and circuit-level analyses using imaging, genetic, and pharmacological approaches to clarify the role of potassium channels in drug use and relapse for improved translational relevance.

## Data Availability

The raw data supporting the conclusions of this article will be made available by the authors, without undue reservation.
