# Supplementary material for: Escalated oxycodone self-administration is associated with expression of voltage gated and calcium activated potassium channels in the mesocorticolimbic system in rats
Source: Front Pharmacol. 2025 Aug 11;16:1653356. doi: 10.3389/fphar.2025.1653356 (PMC12375644; doi:10.3389/fphar.2025.1653356)
Supplement: Supplementary file 1 [file Supplementaryfile1.docx]

**Supplementary table 1 : List of primer sequences used during PCR**

| Channel Type | Gene | Primer Sequence Forward | Primer Sequence Reverse |  |
| --- | --- | --- | --- | --- |
|  |  |  |  |  |
| K_V_ | *Kcna1* | TTG GTA AGG GTG TTC AGA AT | GCA AAG TAC ACT GCA CTA GA |  |
|  | *Kcna2* | TCT CCA TGA CAA CTG TAG GCT ATG | GAC TGG TAA GGC AAT GGT TAA CAC |  |
|  | *Kcna3* | TCA TCT TCT GCT TGG AGA CA | TAT TTC TGG AGA AGG TGG CTT TA |  |
|  | *Kcna4* | AAT CCC CAA CCC CAG GAC CCA A | CTC CAG CTT GCT TCT TCA GGC CA |  |
|  | *Kcna5* | CCC GAT GAT AGA AGT AAT TAA G | TTC TCT AGT ATC CCA GAT GC |  |
|  | *Kcna6* | TAT GGA AGA GAT TCG CTT CTA | GAA CTC TCC GGA TAC TCA AA |  |
|  | *Kcna7* | GGT GTC TCT GTC TCT TTC TCC G | GGT GTC TCT GTC TCT TTC TCC G |  |
|  | *Kcna10* | CCA ATG ACT TTC ACC GGC AAT T | GGC AGA AGA TGG TGA TGG AGA T |  |
|  | *Kcnb1* | CTC CAT CTA CAC CAC AGC AAG T | CTG AAC TTG GGA CTG GTA CTC C |  |
|  | *Kcnb2* | CAC AAC TGT AGG CAA GAC ATT TA | TCC TGG GTT AGA ATG AAT TTC TG |  |
|  | *Kcnd1* | GAA TCT TCA AGT TCT CCA GGC A | AAA GAT GAT GAT AGC CAT GGT |  |
|  | *Kcnd2* | TTG TGA ATG AGC ACA ATG AAA | ATT CAA CTG GCA CAT TAT GTC |  |
|  | *Kcnd3* | GCA ATG GAC TCC TCA ATG AAG C | TTC TAA GCA GTG TAG CAG GTG G |  |
|  | *Kcng1* | AAA GGA TCT GTG TCT CTT AGT | CTT AAA GGT CTG TCT GTT TGC |  |
|  | *Kcng2* | GTA GCC TGG AGG AGA TCG CAA | GGA ATT TCT GGG ACT CAA TTT T |  |
|  | *Kcng3* | CTC TCC GCT GAG TTC CTG AAT T | CCC AGG GAG AAA CAC GTG AAT A |  |
|  | *Kcng4* | TGT CCA CAT ATC CAT GTG TTC | GGT CAC TTT ATT TCA GAT TCG TC |  |
|  | *Kcnq1* | CTA GCT CCC TAA AGC CCC AAA A | GCC AAG TTC TAG TTC CCA CTG A |  |
|  | *Kcnq2* | CTC TAC TCT GGT GAG GAA TAA TC | AAC CGA GGG CTC TAT TAT ATC |  |
|  | *Kcnq3* | TAA GAA GTC TCA GAA AGG GTC AGC | CCA TCA TGC TTT GGT CTT CAG TTT |  |
|  | *Kcnq4* | TGA GAA GGA TGC CAA CTC TGA C | CCA GCC ATG TAT GTG GAG TCT T |  |
|  | *Kcnq5* | TTA AAA GCC TTC AAA CAC GCG T | TCA TCT GTC GTT GTC TCG TGT T |  |
| K_Ca_ | *Kcnma1* | ATG AGA AGC CTG GAT GAC GTT T | TCA GCT TGG CTT GCT CTA TTG A |  |
|  | *Kcnn1* | ACT TCA TGA TGG ACA CAC AGC T | CTT CTT CAC TAG CCT GGT GTG T |  |
|  | *Kcnn2* | CTC AGT CTT CCT AAC CAA GGT CC | ACC AAA ACA ATG GGC ATG ACA TC |  |
|  | *Kcnn3* | GCT TGG TTT GAT CAT CGC CTA C | CTG ATG TAG AGG ATG CGC TCA T |  |
|  | *Kcnt1* | ACC TGT GGG ATT CAG CAC TTA G | CCA GTC TTG GGT GTC ACA TCA |  |
|  | *Kcnt2* | GCT TTG GGG ATG TTA CTC CTG A | GCC AGC TGT TCA AAC TGT ATG G |  |
| Reference Genes | *B2m* | GAT CTT TCT GGT GCT TGT | AGC TCA ATT TCT ATT TGA GGT |  |
|  | *Oaz1* | GCT CAC TCC ATT AGC GG | CCA GAC TTC AAA GGA GG |  |
|  | *Clathrin* | AAG TAT CCG TAA GTC GAG | GGG GTT AAA GTC ACA GAG |  |

**Supplementary Figure 1 : PCR results of Voltage gated Potassium channels that were found to be insignificant in the PFC**
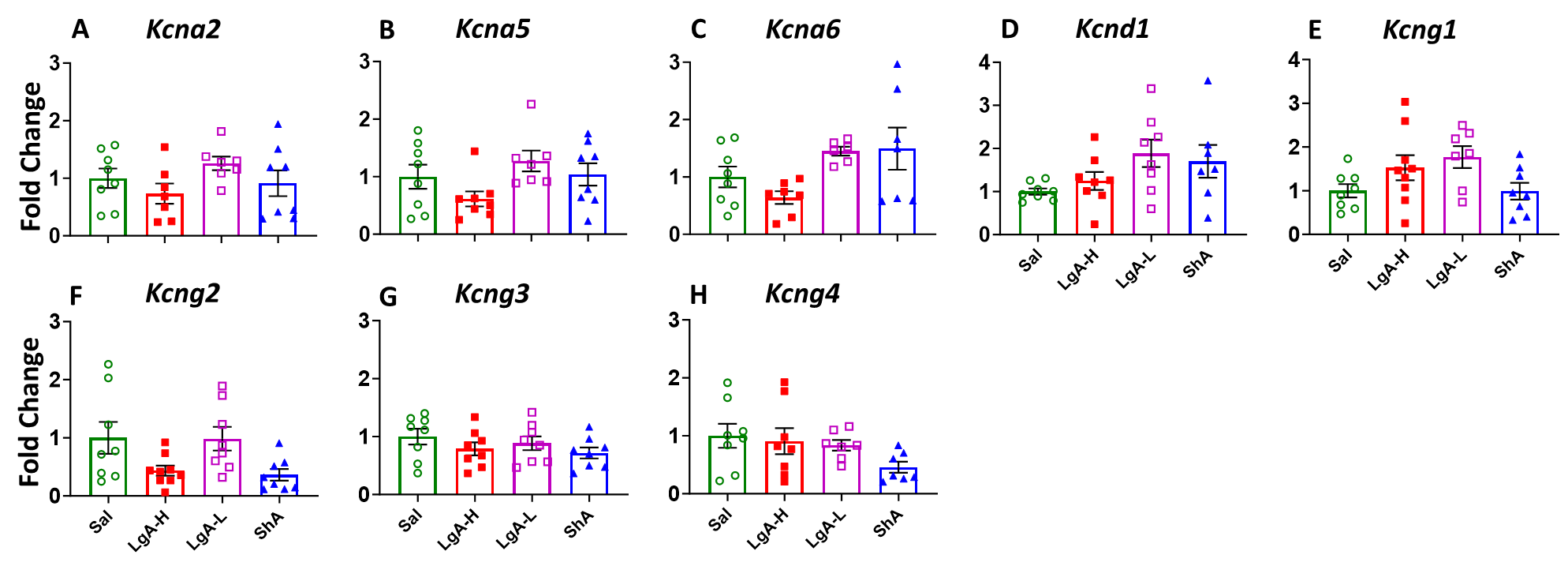


**
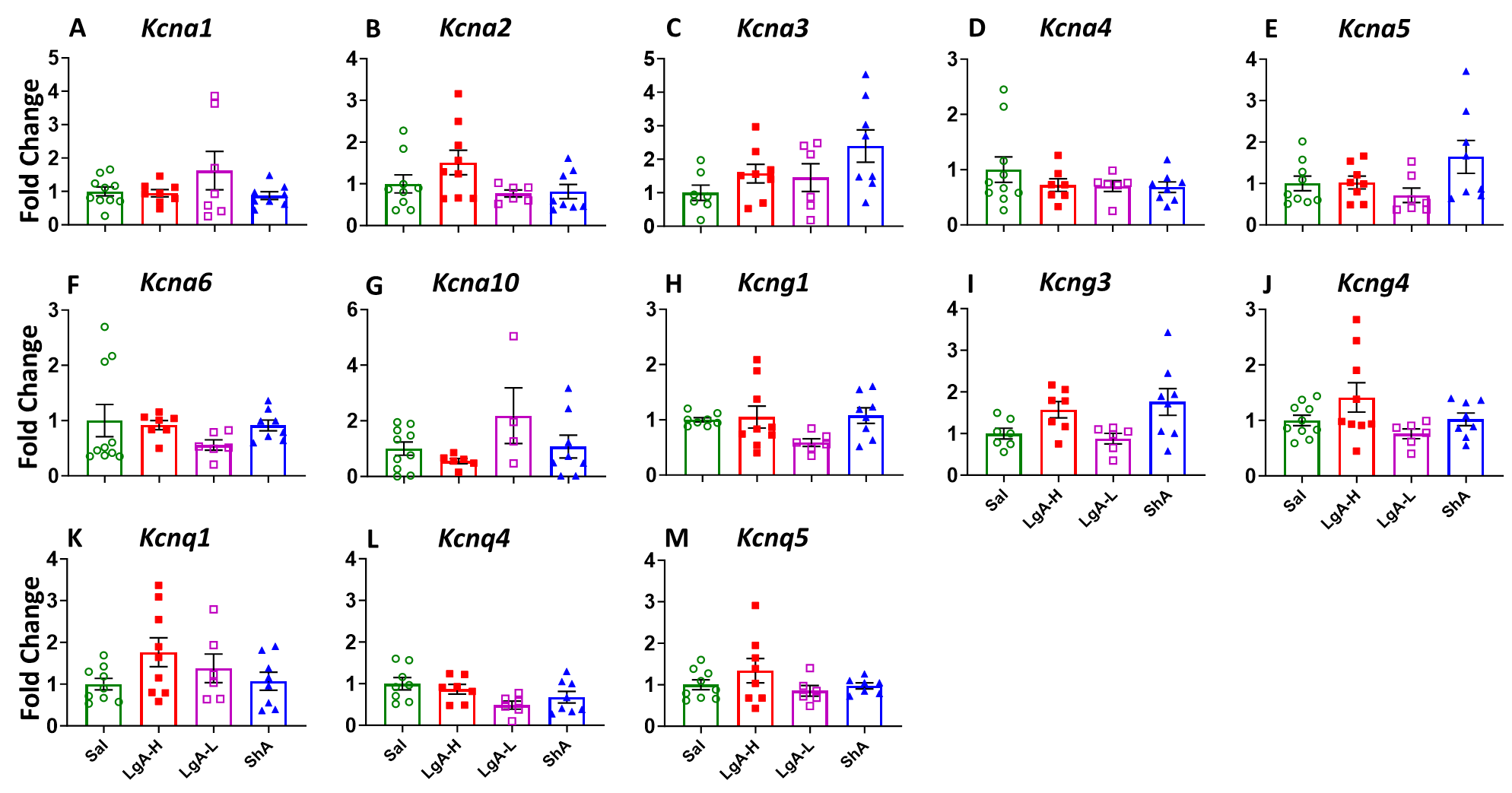
**

**Supplementary Figure 2 : PCR results of Voltage gated Potassium channels that were found to be insignificant in the NAc**

**
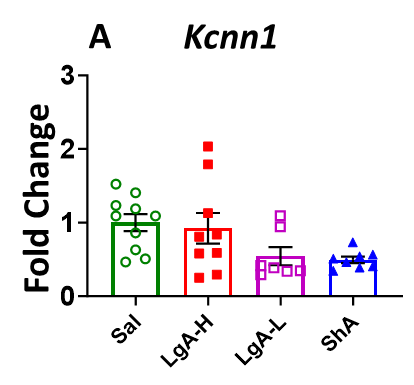
**

**Supplementary Figure 3 : PCR results of Calcium activated Potassium channels that were found to be insignificant in the NAc.**

**
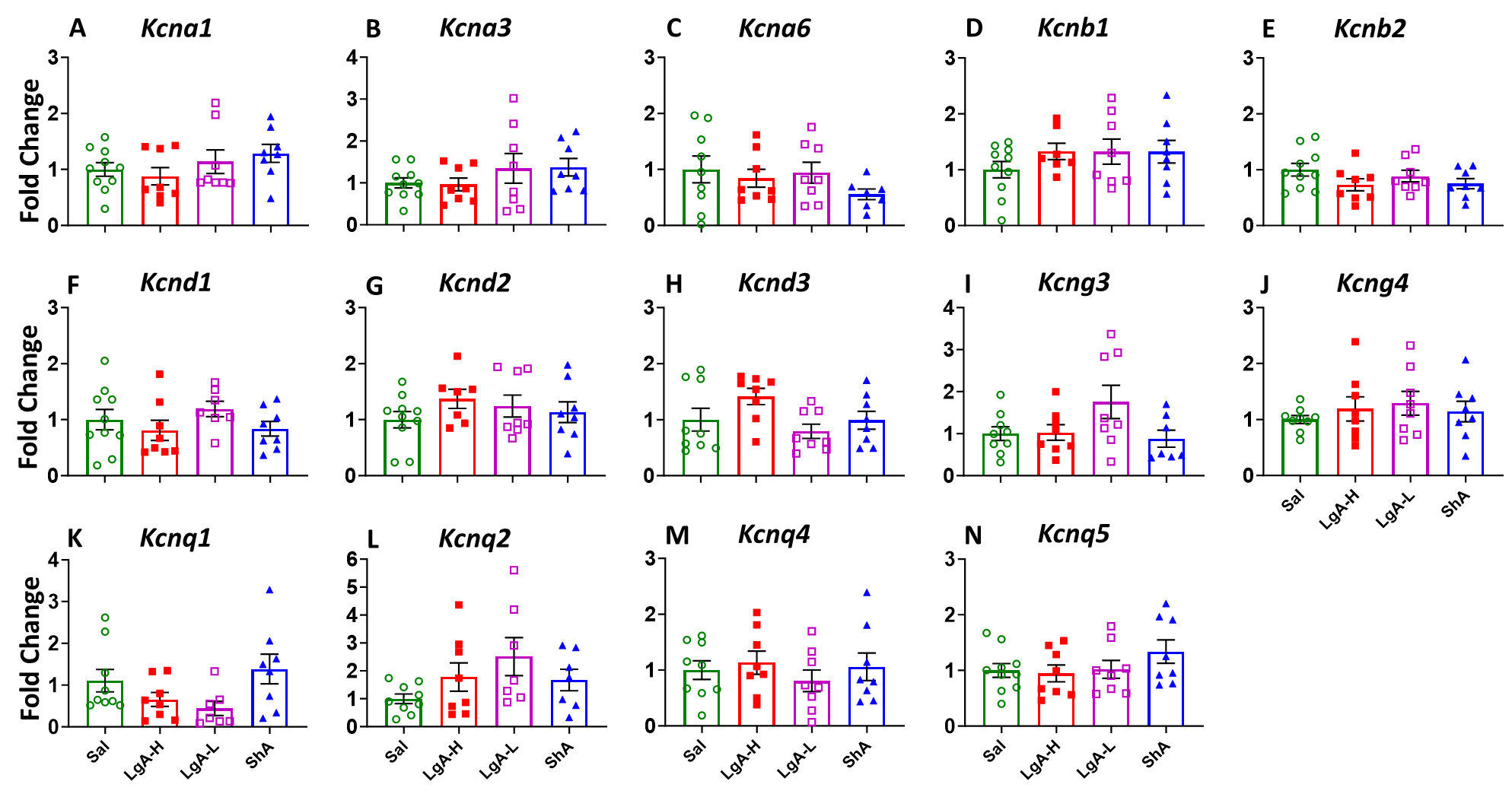
**

**Supplementary Figure 4 : PCR results of Voltage gated Potassium channels that were found to be insignificant in the Hip**


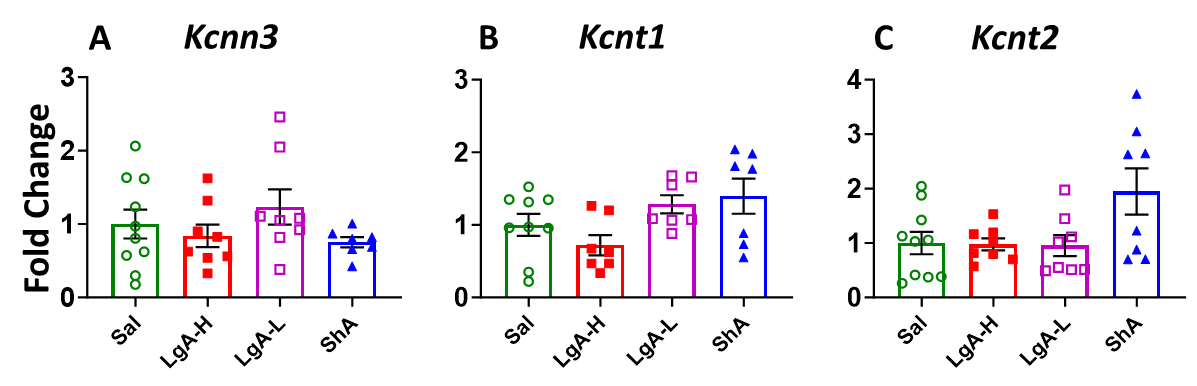


**Supplementary Figure 5 : PCR results of Calcium activated Potassium channels that were found to be insignificant in the Hip**
